# MIROS: A Hybrid Real-Time Energy-Efficient Operating System for the Resource-Constrained Wireless Sensor Nodes

**DOI:** 10.3390/s140917621

**Published:** 2014-09-22

**Authors:** Xing Liu, Kun Mean Hou, Christophe de Vaulx, Hongling Shi, Khalid El Gholami

**Affiliations:** 1 Internet and Information Technology Laboratory, Wuhan University, Road LuoJia, 430072 Wuhan, China; 2 LIMOS Laboratory UMR 6158 CNRS, Blaise Pascal University, Les Cézeaux, BP 10125, 63173, Clermont-Ferrand, France; E-Mails: liu@isima.fr (X.L.); devaulx@isima.fr (C.V.); shi@isima.fr (H.S.); elgholam@isima.fr (K.E.G.)

**Keywords:** operating system, wireless sensor network, real-time, hybrid scheduling, dynamic memory allocation, middleware, multi-core, energy conservation, reliability

## Abstract

Operating system (OS) technology is significant for the proliferation of the wireless sensor network (WSN). With an outstanding OS; the constrained WSN resources (processor; memory and energy) can be utilized efficiently. Moreover; the user application development can be served soundly. In this article; a new hybrid; real-time; memory-efficient; energy-efficient; user-friendly and fault-tolerant WSN OS MIROS is designed and implemented. MIROS implements the hybrid scheduler and the dynamic memory allocator. Real-time scheduling can thus be achieved with low memory consumption. In addition; it implements a mid-layer software EMIDE (Efficient Mid-layer Software for User-Friendly Application Development Environment) to decouple the WSN application from the low-level system. The application programming process can consequently be simplified and the application reprogramming performance improved. Moreover; it combines both the software and the multi-core hardware techniques to conserve the energy resources; improve the node reliability; as well as achieve a new debugging method. To evaluate the performance of MIROS; it is compared with the other WSN OSes (TinyOS; Contiki; SOS; openWSN and mantisOS) from different OS concerns. The final evaluation results prove that MIROS is suitable to be used even on the tight resource-constrained WSN nodes. It can support the real-time WSN applications. Furthermore; it is energy efficient; user friendly and fault tolerant.

## 1. Introduction

With the recent advances in microelectronic, computing and communication technologies, wireless sensor network (WSN) nodes have become physically smaller and more inexpensive. As a result, WSN technology has become increasingly popular in widespread application domains [1,2]. Since WSN nodes are minimized in physical size and cost, they are mostly restricted to platform resources such as processor computation ability, memory resources and energy supply. The constrained platform resources and diverse application requirements make software development on the WSN platform complicated. On the one hand, the software running on the WSN platform should be small in the memory footprint, low in energy consumption and high in execution efficiency. On the other hand, the diverse application development requirements, such as the real-time guarantee and the high reprogramming performance, should be met by the WSN software.

The operating system (OS) technology is significant for the WSN proliferation. An outstanding WSN OS can not only utilize the constrained WSN platform resources efficiently, but also serve the WSN applications soundly. Currently, a set of WSN OSes have been developed, such as the TinyOS [3], the Contiki [4], the SOS [5], the openWSN [6] and the mantisOS [7]. However, many OS development challenges still exist. Firstly, most WSN OSes are either event-driven or multithreaded ones. The event-driven OSes cost less in data memory, but rate poorly in real-time performance. The multithreaded OSes are good in real-time performance but high in data memory cost [8]. Since most WSN nodes are constrained in the memory resources and the real-time guarantee is required by many WSN applications, the development of a WSN OS which is high in real-time performance yet low in memory footprint is a critical challenge. Secondly, the RAM resources on most WSN nodes are precious and need to be utilized efficiently. To utilize the RAM resources efficiently, dynamic allocation needs to be implemented. However, many WSN OSes use the static allocation, such as the TinyOS and the openWSN. With these OSes, the utilization efficiency of the memory resources is low. Some other OSes like the Contiki and mantisOS implement dynamic allocators. However, the allocators are either inflexible or cannot address the memory fragmentation challenge well. Therefore, performance improvement for these allocators is needed. Thirdly, the application programming and reprogramming processes on most current WSN OSes are complicated. The programming is complicated because different application programming patterns are supported by different WSN OSes, thus the users need to understand the diverse low-level system details to begin programming. The reprogramming is complicated because the application part in many WSN OSes is not decoupled from the system part. Thus, the monolithic software image, which can be larger than 100 kilobytes, needs to be updated during the reprogramming process. This process is difficult to complete as the energy resource on the WSN nodes is constrained and the communication bandwidth in the WSN is limited. Therefore, the development of a WSN OS that can on the one hand simplify the application programming complexity, and on the other hand improve the application reprogramming performance becomes essential. Finally, the energy resource on most WSN nodes is constrained. To prolong the WSN nodes' lifetime and avoid the labor work of bringing the deployed nodes back for the power recharging, the energy conservation mechanism needs to be realized. The current sleep/wakeup mechanism used in most OSes for energy conservation is not quite adequate. To reduce significantly energy consumption, a new energy conservation approach becomes essential.

In this article, a new hybrid, real-time, memory-efficient, energy-efficient, user-friendly and fault-tolerant WSN OS MIROS is designed and implemented. MIROS addresses the following challenges: achieving real-time scheduling with low data memory cost; improving utilization efficiency of memory resources; managing energy resource efficiently; simplifying application programming complexity; improving application reprogramming performance; improving node reliability, and developing a new OS debugging approach.

The structure of this article is as follows: In Section 2, the design and implementation of the MIROS hybrid real-time scheduler is presented. With this hybrid scheduling, the number of threads in the MIROS can be decreased. As a result, the real-time scheduling can be achieved with low data memory cost. In Section 3, three kinds of improved MIROS dynamic allocation mechanisms are proposed. These mechanisms are improved on the basis of the current WSN dynamic allocators. With these mechanisms, the allocators become more flexible or can support memory defragmentation. In Section 4, the design and implementation of the new MIROS mid-layer software Efficient Mid-layer Software for User-Friendly Application Development Environment (EMIDE) is discussed. The same as the Embedded Java Virtual Machine (EJVM) and dynamic loading mechanisms (DLM), EMIDE can simplify the application programming process and improve application reprogramming performance. However, it is designed to be both memory and energy efficient, and is thus suitable for use even on severely resource-constrained WSN nodes. In Sections 5 and 6, the works about using the multi-core hardware platform to reduce energy consumption and improve node reliability are investigated, respectively. In Sections 7 and 8, the MIROS inter-process communication (IPC), the new OS debugging method and the ongoing studies are presented.

## 2. Hybrid Real-Time Scheduler in the MIROS

In this section, the design and implementation of the new MIROS scheduler which can achieve the real-time scheduling with less data memory cost is presented.

### 2.1. Scheduling Background of the Wireless Sensor Network Operating Systems (WSN OSes)

The scheduling models can be classified into two kinds: the multithreaded scheduling and the event-driven scheduling. Multithreaded scheduling is commonly used on the general-purpose OSes, such as Linux and uCOS. Different tasks can be executed concurrently by the threads, and the preemption among the threads can be achieved by the context switch. With the preemption support, the real-time (RT) reaction can be achieved. However, each thread in the preemptive multithreaded OS should have an independent stack. Thus, the data memory usage of the preemptive multithreaded OS is high, making the preemptive multithreaded OS unsuitable for the high resource-constrained WSN nodes, e.g., the MicaZ node which has only 4 KB RAM. To address this challenge, the event-driven scheduling is developed. Different from the preemptive multithreaded OS, each task in the event-driven OS should run to completion before the execution of the next task. As a result, only one stack is needed in the event-driven system and a considerable amount of data memory can be saved if compared to the preemptive multithreaded system. In article [9], the results show that the event-driven OS can achieve 30-times improvement in data memory consumption over the general-purpose multithreaded OS. This is significant for the resource-constrained WSN platforms. However, the deadline of real-time tasks cannot be guaranteed in the event-driven OS due to the non-support of the preemption. As a result, the event-driven OS is not suitable to be used for real-time WSN applications.

Two scheduling models have different strong and weak points. The design and implementation of a new OS scheduler that cannot only support the real-time scheduling, but also consume less data memory will be a key research challenge for the WSN OS.

### 2.2. Motivation of the Hybrid Scheduler in MIROS

To address the challenge of running the multithreaded OS on the memory-constrained WSN nodes, the thread-stack optimization technique *stack-size analysis* [10] is developed. With this technique, the worst-case thread-stack usage can be computed at the compile time, and then each stack can be allocated to a minimal but system-safe size during the run-time. By doing this, the data memory consumed by the multithreaded OS can be reduced.

*The stack-size analysis* approach can reduce the size of each thread stack in the multithreaded OS. In addition to this approach, another method to decrease the data memory usage of the multithreaded system is to decrease the total number of the thread stacks. Currently, this approach has been used in MIROS, and it is achieved by the MIROS hybrid scheduler.

### 2.3. MIROS Hybrid Scheduling Structure

In MIROS, the hybrid scheduling is developed for the purpose of combining the advantages of both event-driven scheduler's low memory consumption and multithreaded scheduler's high real-time performance. Firstly, the event-driven scheduling is implemented to keep the low data memory consumption. However, the real-time performance of the event-driven scheduling is poor as the preemption is not enabled. Thus, the multithreaded scheduler is also implemented and it is used dedicatedly to schedule the RT tasks. Consequently, the hybrid scheduling (both the event-driven scheduling and the multithreaded scheduling) is realized in MIROS. With this hybrid scheduling, the real-time OS can be achieved and the OS data memory usage is not high.

**Figure 1.**
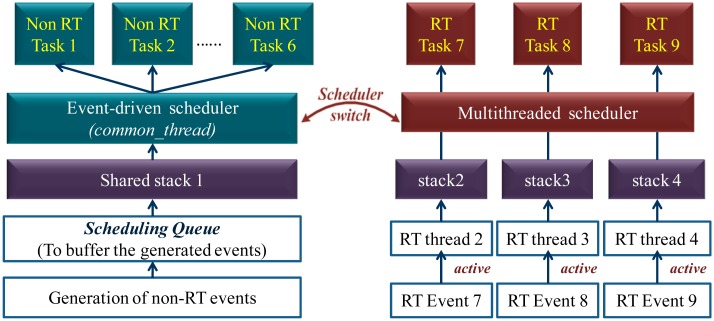
Hybrid scheduling structure in the MIROS.

The hybrid scheduling structure of MIROS is depicted in Figure 1. Both the event-driven scheduler and the multithreaded scheduler are implemented in parallel. The system tasks are classified into two kinds: the RT ones and the non-RT ones. The RT tasks are scheduled by the multithreaded scheduler while the non-RT tasks are scheduled by the event-driven scheduler. Two schedulers can switch to each other, but only one scheduler can be active at any time. The multithreaded scheduler has the priority higher than the event-driven one, thus it can preempt the event-driven scheduler whenever necessary. In case all the RT tasks are inactive, the OS will run in the pure event-driven scheduling model. However, if any RT task becomes active, the event-driven scheduler will be suspended, and then the OS will switch to the multithreaded scheduling model. When running in the multithreaded scheduling model, the *RMS* (Rate Monotonic Scheduling) [11] scheduling algorithm is applied. This algorithm is chosen because it uses the static priority scheme. Thus, it is simple to implement and the execution overhead is low. Since the computation ability and the memory resources of the WSN nodes are constrained, the applying of this algorithm is appropriate.

The advantage of this MIROS hybrid scheduling is that a RTOS can be achieved with low data memory consumption. It is assumed that there exist nine tasks *T_1_*, *T_2_*, *…*, *T_9_* and three of them (*T_7_*, *T_8_*, *T_9_*) are RT ones (Figure 1). If the pure multithreaded OS (mantisOS, uCOS, *etc.*) is used to schedule these tasks, nine threads along with nine stacks need to be created, as in the pure multithreaded OS one thread will be created for the execution of one task whether or not this task is RT (the difference may be that the RT task is executed by a high-priority thread while the non-RT task is executed by a low-priority one). However, the non-RT tasks have loose constraint in the response time, and the preemption is not needed among their executions. Thus, they can be scheduled one after one by the event-driven scheduler. Consequently, only four stacks rather than nine need to be allocated: three of them are for the multithreaded scheduler to execute the three RT tasks (*T_7_*, *T_8_*, *T_9_*), while the left one is for the event-driven scheduler to execute all the non-RT tasks (*T_1_* to *T_6_*). As a result, the number of the stacks in MIROS can be decreased greatly. Since the size of each thread stack is commonly large, e.g., in mantisOS each stack is set to 120 bytes, the data memory usage in the MIROS becomes smaller if compared with that in the pure multithreaded WSN OSes.

### 2.4. Implementation of the MIROS Hybrid Scheduler

In this section, the implementation of the MIROS hybrid scheduler and the scheduler switch mechanisms are presented.

**Figure 2.**
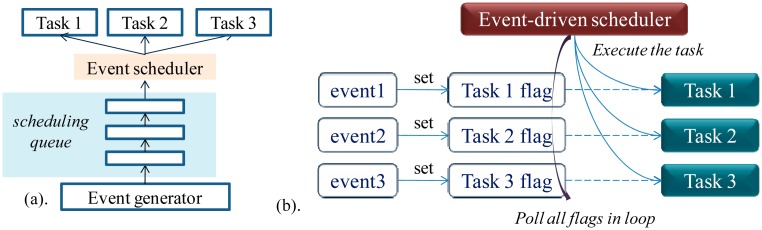
Event-driven scheduling structure. (**a**) General event-driven scheduling structure. (**b**) MIROS event-driven scheduling structure.

*Implementation of MIROS event-driven scheduler*: In most event-driven WSN OSes, the scheduling queue mechanism is used for the implementation of the event-driven scheduler. In these OSes, the generated events are buffered in the scheduling queue, and then extracted and dispatched one by one by the event scheduler, as seen in Figure 2a. The scheduling queue is needed as the event-generating speed can be quicker than the event-dispatching speed. The scheduling principle can be the First Input, First Output (FIFO) or priority-based; the latter one supports real-time performance better.

MIROS event-driven scheduler is typical in that only the non-RT tasks need to be schedule by it. Thus, the real-time performance is not a critical design factor, and the flag-polling mechanism other than the scheduling queue mechanism is used to schedule the events. In Figure 2b, the structure of the MIROS event-driven scheduling is depicted. Every non-RT task in the MIROS has one-bit flag. Once a task is activated, the related task's flag will be set. The event scheduler will poll the flags in loop. If a flag is found to be set, the related task will be executed. Task flags have the priorities that are statically assigned offline. The flags with the higher priorities will be polled in advance, thus the corresponded tasks can be executed earlier. If no flags are set, the sleeping directive will be executed, and then the node will fall asleep to conserve the energy. Compared with the scheduling queue mechanism, this flag-polling mechanism is less flexible as the scheduling sequence is fixed, but it is more efficient and more suitable for the non-RT task scheduling in the MIROS.

*Implementation of MIROS multithreaded scheduler*: Thread management is a key topic for the implementation of the multithreaded scheduler. It is essential as the number of the threads in the multithreaded OS can be large, e.g., in the uCOS the thread number can be as large as 64 or even 256. To schedule these threads efficiently, a sound thread management mechanism is needed. Currently in the uCOS and the mantisOS, the “8 × 8” lookup table [12] and the multilevel thread queues [7] are used respectively for the purpose of managing the threads efficiently.

MIROS is different from the pure multithreaded OSes (uCOS, mantisOS, *etc.*) in that only the RT tasks need to be executed by threads, thus the thread number is small and no complicated thread management mechanism is needed. As a result, a simple single-link queue is used for the thread management. In this queue, all the MIROS threads are queued in the order of their priorities (from highest priority to the lowest priority). The next thread to be scheduled can then be determined quickly by finding the first active thread in this thread queue.

*Scheduler switch in MIROS*: In MIROS, two kinds of switches exist. One is the *thread switch* which occurs within the multithreaded scheduling system, and the other is the *scheduler switch* which occurs between the event-driven scheduler and the multithreaded scheduler. In order to make the switching process efficient and easily managed, the event-driven scheduler in the MIROS is also implemented as a thread, named the “*common_thread.*” The *run* function of this *common_thread* is to execute the MIROS event-driven scheduler. By doing this, the MIROS hybrid scheduling can be managed as the pure multithreaded scheduling: (1) If all the RT threads are inactive, the next thread to be scheduled will be the *common_thread*. In this case, the OS will switch to the event-driven scheduling model, and all the non-RT tasks will be scheduled one by one. (2) If the *common_thread* is executing and any RT thread becomes active, the *common_thread* will be preempted, and then the OS will switch to the multithreaded scheduling model. In so doing, the hybrid scheduling in MIROS can be implemented simply and executed efficiently.

### 2.5. Related Works about the Hybrid Scheduling in the WSN OSes

Currently, the hybrid scheduling structure has been implemented in several WSN OSes in order to address the different kinds of WSN challenges.

**Figure 3.**
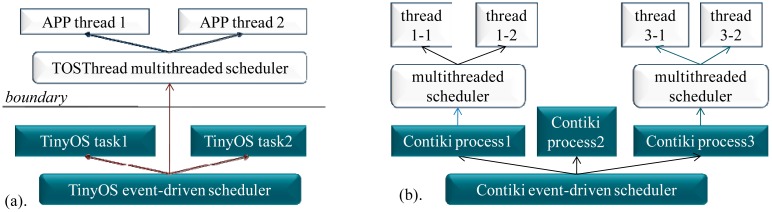
(**a**) Hybrid scheduling structure in the TinyOS TOSThread. (**b**) Hybrid scheduling structure in the Contiki multithreading.

In TinyOS, the TOSThread [13] is developed for the purpose of combining the *ease* of the threaded programming model with the *efficiency* of a fully event-driven OS. TOSThread is achieved in the TinyOS application level (Figure 3a). It supports the application logic to be implemented in the user-level preemptive threads, and supports the lengthy computation program to be developed inside the TinyOS application.

In Contiki [4], the hybrid scheduling structure is also implemented (Figure 3b). The same as the TOSThread in the TinyOS, the multithreaded scheduler in the Contiki is also built on top of the event-driven scheduler. However, differing from TOSThread, the multithreading in the Contiki is not used expressly for the user application. Instead, it is applied optionally in the low-level system, and serves the Contiki *processes* that need the preemption support (e.g., the lengthy computation task).

LIMOS [14] is another hybrid WSN OS. The LIMOS multithreaded scheduler is built on top of the event-driven scheduler, as well. However, as opposed to the former two mechanisms, the scheduling model in the LIMOS can be configured in terms of the run-time contexts: (1) In the case that the thread number inside each process is configured to be 1, LIMOS runs in the pure event-driven scheduling model. (2) In the case that the process number in the system is configured to be 1, LIMOS runs in the pure multithreaded scheduling model. As a result, LIMOS becomes customizable and context aware, and can thus adapt flexibly to different application contexts.

The hybrid scheduling structure in the MIROS is different from the mechanisms above in that the MIROS multithreaded scheduler is implemented in parallel with the event-driven scheduler (Figure 1), and the design purpose of this hybrid scheduling is to achieve the RTOS with low data memory consumption. A key drawback of the hybrid scheduling structure in the TinyOS, Contiki and LIMOS is that these OSes are still not real-time ones even if the hybrid scheduler is implemented. This is because the event-driven scheduling is still used in the native scheduling layer of these OSes. For example, if the Contiki *thread 1-2* is executing and the *thread 3-1* needs to be executed quickly (Figure 3b), the preemption from the *thread 3-1* to the *thread 1-2* cannot be achieved immediately. This is because all the Contiki *processes* are still scheduled by the event-driven scheduler, and the *process 3* can be executed only after the *process 1* runs to completion.

### 2.6. Performance Evaluation

**Figure 4.**
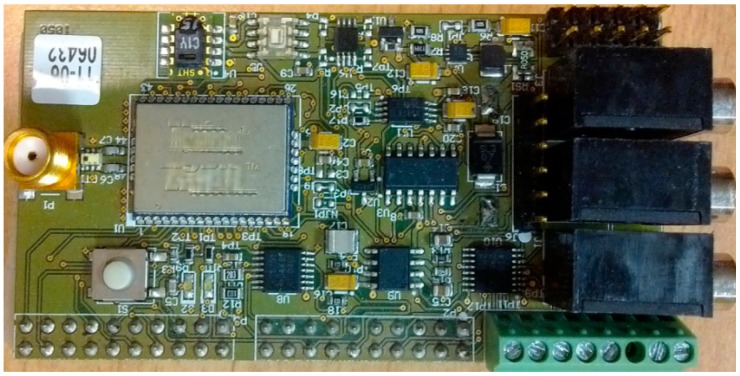
iLive node. ILive is equipped with the AVR ATmega1281 microcontroller (128 KB FLASH, 8 KB RAM), one temperature sensor, one light sensor, one air sensor, one humidity sensor, three soil moisture decagon sensors [16] and four soil moisture watermark sensors [17].

In this section, the memory consumption and the execution efficiency of the schedulers in the TinyOS, Contiki, SOS, mantisOS and MIROS are evaluated. The evaluation is done on the iLive node (Figure 4). The AVR studio [15] is used as the software development tool.

#### 2.6.1. Code Size of the Different OS Schedulers

**Figure 5.**
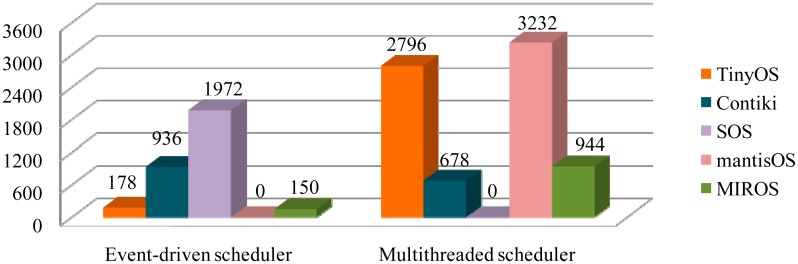
Code size of the event-driven and multithreaded schedulers in the different OSes (bytes).

*Code size of the event-driven schedulers:* The code sizes of the different event-driven schedulers are shown in Figure 5. In the *TinyOS*, only one FIFO scheduling queue is used for the event scheduling, and the scheduler code size is small. In the *Contiki*, two level scheduling mechanisms are adopted: the FIFO scheduling queue for the asynchronous events and the polling mechanism for the high-priority events. As a result, the scheduler code size is larger than that in the TinyOS. In the *SOS*, three priority-based scheduling queues are used. Moreover, the indirect access and the module management mechanisms are implemented [5]. Consequently, the SOS scheduling structure is more complicated and more code memory is consumed. In the *MIROS*, the simple flag-polling mechanism is applied and the code size is small. In *mantisOS*, no event-driven scheduler is implemented.

*Code size of the multithreaded schedulers:* The code size of the different multithreaded schedulers is shown in Figure 5. The *mantisOS* scheduler consumes more code memory than the others. This is because mantisOS is a pure multithreaded OS, and all the tasks in this OS are executed by threads. As a result, the thread number is large. In order to manage these threads efficiently, the multilevel-queue (five ready queues and one sleep queue) scheduling mechanism is implemented, and this increases the complexity of the mantisOS scheduling architecture. In the *MIROS*, only the RT tasks need to be scheduled by the multithreaded scheduler, thus the thread number is small and the multithreaded scheduling structure is simple (Section 2.4). In consequence, the code memory consumption is not high. In the *TOSThread* [13], the code size of the multithreaded scheduler is large; this is because a flexible boundary between the user code and the kernel code is implemented in TOSThread. With this approach, the TinyOS core can keep unchanged, thread-safe and non-invasive. However, the code size of the TOSThread becomes larger.

#### 2.6.2. Data Memory Consumption of the Different OS Schedulers

The data memory consumption of the event-driven scheduler can be evaluated as follows:

(1) $$E_{1}=\text{Size}(\text{DATA}/\text{BSS})+\text{Size}(\text{PCB})+\text{Size}(\text{SQ})=M_{\text{DATA}/\text{BSS}}+S_{\text{PCB}}*N_{\text{PCB}}+S_{\text{SQ}}*L_{\text{SQ}}$$

in which *M_DATA/BSS_* represents the size of the DATA/BSS sections (the global variables, static variables, *etc.*). *S_PCB_* and *N_PCB_* represent respectively the structure size of the *process control block* (in SOS, it is named as *module control block*) and the number of the defined *processes. S_SQ_* and *L_SQ_* represent respectively the size of each entry in the scheduling queue (SQ) and the queue length of the SQ.

The data memory consumption of the multithreaded scheduler can be evaluated as follows:

(2) $$E_{2}=\text{Size}(\text{DATA}/\text{BSS})+\text{Size}(\text{TCB})+\text{Size}(\text{STK})=M_{\text{DATA}/\text{BSS}}+S_{\text{TCB}}*N_{\text{TCB}}+S_{\text{STK}}*N_{\text{THRD}}$$

in which the *S_TCB_*, *N_TCB_*, *S_STK_* and *N_THRD_* represent respectively the structure size of the *thread control block* (TCB), the number of the thread TCBs, the size of the thread stack and the number of the allocated threads.

In Table 1, the date memory consumption of the different schedulers is listed. In the *MIROS*, the flag-polling mechanism is used for the event-driven scheduling, thus no scheduling queue exists. In *TinyOS*, *Contiki* and *MIROS*, the multithreading is used either only for the application (TinyOS), or only for the system preemption-needed *processes* (Contiki), or dedicated to the real-time tasks (MIROS). Therefore, the number of the threads and the TCB in these OSes is smaller if compared to those in the pure multithreaded OS mantisOS. Provided by a scenario shown in Table 1, the data memory size of the different OS schedulers can be computed.

From the results in Table 1, it can be concluded that the MIROS hybrid scheduler can support real-time scheduling by consuming less data memory resources (compared to the pure multithreaded OS mantisOS). Although the data memory costs of the TinyOS TOSThread and the Contiki multithreading are also not high, the real-time scheduling cannot be guaranteed in these systems (Section 2.5).

From the results in Figure 5 and Table 1, it can be concluded that the code memory size and the data memory size of the SOS scheduler is larger if compared with those of the other event-driven OSes; this is because SOS is a dynamic module-based OS. In SOS, the complicated module management and indirect access mechanisms need to be implemented.

#### 2.6.3. Execution Efficiency of the Different OS Schedulers

The execution efficiency of the scheduling primitives can be evaluated by the clock cycles, and the evaluation results are shown in Tables 2 and 3.

In MIROS, the simple flag-polling mechanism is used for event scheduling, thus the clock cycles of the event posting and the task dispatching are small. As for the thread scheduling, the static priority-based RMS algorithm is implemented. The clock cycles to select the next thread is (5 + 9n), where n represents the searching steps in the thread queue. In the case that n is 3 (thread number is small in MIROS, Section 2.4), the clock cycle will be 32.

#### 2.6.4. Discussions

Although two schedulers are implemented, the code size of the MIROS does not greatly increase. This is because the MIROS event-driven scheduler only needs to schedule the non-RT tasks while the MIROS multithreaded scheduler only needs to schedule the RT tasks. As a result, the implementations of both the two schedulers can be simplified (Section 2.4).

The MIROS RT tasks and non-RT tasks are scheduled by two different schedulers. Thus, two kinds of programming styles exist. Commonly, the non-RT tasks need to be programmed by the event-based style (using the phase-split state machine) while the RT tasks need to be programmed by the thread-based style. In order to simplify the programming complexity, the protothread [18] is applied in MIROS. With the protothread, the non-RT tasks can also be programmed in a thread-similar programming style. In this way, the programming differences between these two kinds of tasks can be eased.

As discussed in the Section 2.3, the MIROS event-driven tasks (non-RT tasks) have the priorities lower than the thread tasks (RT tasks), thus they can be preempted by the latter ones. However, if an event-driven task is preempted by a thread task, and this event-driven task has taken up a resource which is needed for this thread task to continue its execution, the priority inversion problem will occur. To solve this problem in this case, the priority of the event-driven task needs to be improved so that this task can be executed preferentially and release the shared resource that it has taken up quickly.

Optionally, the MIROS multithreaded scheduler can be developed as a configurable component. If the real-time requirement is not needed by the applications, this component does not need to be built. In this case, the code size of the MIROS can be reduced further.

## 3. Memory Management in MIROS

Memory management is important for the WSN as it can help to utilize memory resources more efficiently. Static allocation has been used in several WSN OSes, such as the TinyOS and the openWSN. This approach has low execution overhead. However, it is not flexible to adapt to the varied WSN contexts. Moreover, the memory resources cannot be utilized efficiently. To improve the allocator flexibility as well as the memory utilization efficiency, the dynamic allocation needs to be implemented.

Buddy memory allocation [19,20] is a popular representative of the dynamic allocation. This approach is relatively easy to be implemented and has fast allocation response time. Moreover, it supports efficient splitting and coalescing of the memory blocks. However, it has the drawback of producing large internal fragmentation. In the article [21], it is concluded that the fragmentation percentage of the buddy systems can be up to 50%. As a result, this approach is not fit for use in memory-constrained WSN nodes. Besides the buddy systems, the sequential fits (SF) and the segregated free lists (SFL) are the two basic dynamic allocation mechanisms, and they are commonly realized in the current WSN OSes.

### 3.1. Dynamic Memory Allocators in the Current WSN OSes

**Figure 6.**
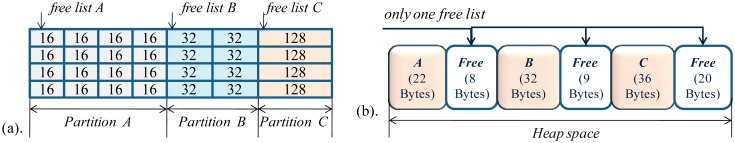
(**a**) SOS segregated free list (SFL) allocation. Three partitions (*A*,*B*,*C*) are pre-reserved. The block allocation is applied in each partition, and the block sizes in the different partitions are different. (**b**) mantisOS sequential fit (SF) allocation. Only one double-link free list is used to manage all the free memory.

Segregated free list (SFL) allocator has been used in Contiki, SOS and uCOS. It divides the memory heap into segregated partitions. Each partition holds a set of fixed-size blocks, and a free list is used for the block allocation. Upon allocation, a block is deleted from the header of the matched free list. Upon releasing, the released block is added to the header of the matched free list. In Figure 6a, the SOS SFL allocation mechanism is depicted.

Sequential fit (SF) allocator is different from the segregated free list (SFL) allocator in that the memory heap is not divided into segregated partitions. Instead, all the allocations are performed inside the heap in a sequential way, and only one free list rather than several segregated free lists is used to manage the free memory. Upon allocation, the free list is searched to find a suitable free entry, and the searching algorithm can be the best fit, the first fit, the next fit or the good fit [22]. In Figure 6b, the mantisOS SF allocation mechanism is depicted.

The SFL allocator shows its advantage in the short and deterministic allocation time. This feature is significant for the real-time systems. However, each SFL partition needs to be pre-reserved. If the pre-reserved size is too small, the memory overflow problem will occur. If it is too large, the memory utilization efficiency will be low and the memory insufficiency problem can occur. Due to these reasons, the SFL allocator is not flexible to adapt to the varied application contexts.

The key advantages of the SF allocator are that no memory area needs to be pre-reserved and no internal memory fragmentation exists. However, the allocation time is not deterministic as an appropriate entry needs to be searched upon the allocation. Moreover, the external memory fragmentations exist, e.g., in Figure 6b, if a 30-byte object needs to be allocated, the allocation will fail although the total size of the free memory is larger than 30 bytes. Currently in the mantisOS, no mechanism is implemented to solve this fragmentation problem, and this will decrease the utilization efficiency of the memory resources.

### 3.2. Dynamic Memory Allocators in MIROS

To address the challenges presented above, the *heap-extendable SFL allocator* and the *defragmented SF allocator* are developed in MIROS. With these allocators, the flexibility problem of the *SFL* allocator can be eased, and the fragmentation problem of the *SF* allocator can also be solved.

#### 3.2.1. MIROS Heap-Extendable Segregated Free List Allocation

**Figure 7.**
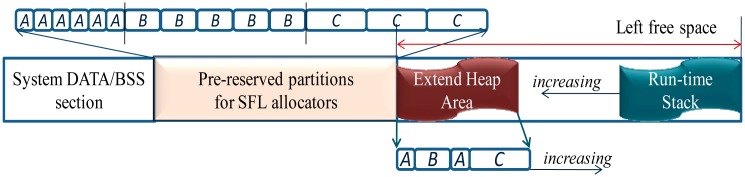
Heap-extendable segregated free list (SFL) allocation in the MIROS. After the partitions are pre-reserved, the left memory space will be utilized both for the extended heap and the run-time stack.

To improve the flexibility and ease the pre-reservation problem, the heap extending mechanism is applied in the MIROS SFL allocator. With this mechanism, no partition needs to be reserved to the maximum size, but to a moderate size which will be enough for most run-time contexts. In case that a partition overflows, the allocation will not fail but continues in the extended heap area, as seen in Figure 7. In so doing, the MIROS SFL allocator becomes flexible. This flexibility feature is important for the WSN as the WSN contexts are diverse and it is impossible to find an ideal pre-reserved solution which will be suitable for all the diverse WSN contexts, e.g., if a WSN node is configured to be a router, the size required by the *packet buffering* partition will be greatly different from the case when this node is configured to be an end-device. However, after the MIROS heap-extendable strategy is applied, this problem can be eased.

#### 3.2.2. MIROS Defragmented Sequential Fit Allocation

To solve the fragmentation problem in the *SF* allocation, the defragmentation *mechanism* is applied in the MIROS. Currently, two defragmentation approaches are realized: the *proactive* defragmentation and the *reactive* defragmentation (concept motivated by the *proactive* routing protocol and the *reactive* routing protocol).

**Figure 8.**
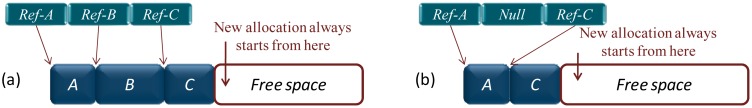
MIROS proactive defragmentation *SF* allocation. After *B* is de-allocated in Figure 8a, the *C* will be assembled with *A* proactively. Thus, no fragments will appear and the free memory space is continuous.

*MIROS proactive defragmented SF allocator:* With this mechanism, the memory fragments will be assembled proactively once they appear, and no fragments will exist in the heap (Figure 8). The advantage of this approach is that the new allocation can be performed immediately from the starting address of the free space and the allocation time is short and deterministic. However, the addresses of some objects will change after the memory is coalesced, thus each allocated object needs to be accessed indirectly by means of the reference pointer. After an object has moved to a new address, moreover, the update to its reference pointer can help it remain accessible.

**Figure 9.**
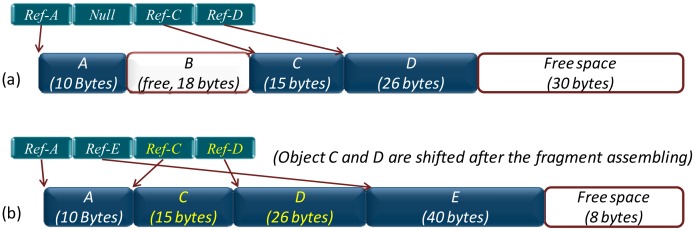
MIROS reactive defragmentation sequential fit allocation. If a 20-byte object needs to be allocated in Figure 9a, the defragmentation operation is not needed. However, if a 40-byte object *E* needs to be allocated, the defragmentation operation will be performed (Figure 9b), as there is not enough continuous free memory in this case.

*MIROS reactive defragmented SF allocator:* The *reactive* mechanism is different from the *proactive* mechanism in that the fragments are not assembled every time an object is released. Instead, they are assembled only when the first time allocation is failed, that is, when there is not enough continuous free memory existing in the heap (Figure 9). In order to avoid the redundant assembling operations, not all the fragments are assembled during the defragmentation process. Instead, only parts of the fragments are assembled and the assembling operation will stop once enough continuous memory has been produced for the current allocation. The same as the *proactive* mechanism, the addresses of some objects will change after the fragments are assembled, thus the reference pointers need also to be used in this *reactive* mechanism.

### 3.3. Performance Evaluation

**Figure 10.**
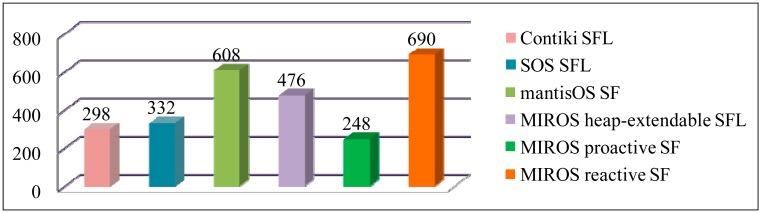
Code size of the different dynamic memory allocators.

In Figure 10 and Table 4, the code size, the execution efficiency and the memory utilization efficiency of the different dynamic allocators are evaluated. In Table 4, the symbol *F* represents the steps of searching for a free block in the Contiki SFL allocator (Contiki SFL uses the block flag mechanism other than the free list mechanism to manage the free memory), *L* represents the steps of searching for an available entry in the free list, and *S* represents the size of the memory to be shifted during the coalescing operation.

From these evaluation results, it can be concluded that the code size and the allocation overhead of the dynamic allocators are not very high, thus the dynamic allocation approach is feasible to be used even on the resources-constrained WSN platforms. This result has also been proved in the article [23]. In this article, the authors insisted that the costs of the dynamic allocation are simply overestimated.

Different allocation mechanisms have different strong points and weak points, and they strike the tradeoff among the allocation response time, the execution overhead and the memory utilization efficiency. The allocation response time is concerned with the real-time performance, the execution overhead is concerned with the energy cost, and the memory utilization efficiency is concerned with the memory constraint problem. As for which approach should be selected, it depends on the practical contexts. If the memory resource on the WSN platform is not highly constrained (e.g., the SunSPOT node which has 512 KB RAM) and the real-time tasks exist in the system (e.g., the periodic signal sampling tasks), the MIROS SFL approach can be an ideal choice. This is because the SFL approach has fast allocation response time and low allocation and de-allocation overhead, thus the real-time requirements can be better satisfied and it will cost fewer energy resources. However, if the memory resource on the nodes is precious (e.g., the Mica2 node with only 4 KB RAM) and the application tasks have a loose requirement to the real-time guarantee (e.g., the precise agricultural application in which the nodes only need to sample the environmental data several times a day and transmit the sensing packets to the base station), the MIROS reactive SF approach can be used. With this approach, the memory fragments can be assembled when needed. Consequently, the allocation failure resulting from the memory insufficiency problem can be eased. As for the MIROS proactive SF approach, its drawback is high de-allocation overhead, but its advantage is fast allocation time and defragmentation support. Therefore, it is suitable to be used for the real-time WSN applications in which the nodes are constrained in the memory resources (e.g., the MicaZ) but sufficient in the energy resources.

Nevertheless, the design and implementation of a new allocation mechanism that is fast in allocation time, low in allocation overhead, yet high in memory utilization efficiency still requires further study.

## 4. Mid-Layer Software EMIDE for User-Friendly Application Development Environment

Programming and reprogramming are the two important processes for application development in the WSN. Currently, these processes are difficult for the WSN users. On one hand, the hardware platforms in the WSN are diverse, thus the users need to understand the diverse low-level hardware details to make the application programming. On the other hand, most WSN nodes are prone to be deployed in the harsh environments where humans cannot access, thus the WSN reprogramming needs to be done remotely through the wireless. Yet, the wireless transmission in the WSN is high in energy consumption and limited in communication bandwidth [24]. All these features make the WSN reprogramming process complicated.

One way to address the challenges above is to decouple the application space from the low-level system space and provide a set of services and programming interfaces in the system space. In this way, the software space will be divided into two parts: the application space and the system space. Subsequently, two separated images will be generated: the application image and the system image. The system image can be built by the WSN experts and pre-burned to the WSN nodes. The WSN users then only need to focus on the application space and upload the application image to the target nodes when necessary. Through these means, application programming can be simplified as the users are no longer needed to understand the low-level system details. Moreover, the reprogramming performance can be improved as only the application image other than the monolithic software image needs to be updated.

To decouple the user application from the WSN system, two kinds of mechanisms are commonly used. One is the usage of the EJVM (Section 4.1), and the other is the usage of the DLM (Section 4.2). Besides these two mechanisms, the new approach EMIDE, which is a mid-layer software in MIROS, is also developed and presented in the Section 4.3.

### 4.1. Embedded Java Virtual Machine (EJVM)

EJVM is encouraged for use in the WSN due to the following reasons. Firstly, after the EJVM is applied, the users can program the WSN application by the popular, reusable and robust Java language without the necessity of considering the low-level platform details. Secondly, Java is an object-oriented paradigm, thus the WSN application design, test and maintenance process can be simplified. Thirdly, the Java byte code is platform independent, thus the challenge of programming in the heterogeneous WSN environments can be addressed [25]. Finally, the WSN reprogramming performance can be improved as only the Java application image needs to be updated during the reprogramming process.

Currently, a set of EJVMs have been developed, including the TinyVM [26], the Darjeeling VM [27], the simpleRTJ [28], the SquawkVM [29], the nanoVM [30], the Jwik [31] and the Java Card VM [32]. Different JVMs have different features and strike a tradeoff between the VM performance and the resource consumption (Table 5). For all the EJVMs listed in Table 5, the pre-linked mechanism is used for the produce of the application image. After the Java application programs are built, all the separated Java classes will be pre-linked statically to form a single image. Compared to the general-purpose JVMs in which all the classes are loaded dynamically during the run-time, the pre-linked image is less flexible, but it is more appropriate for the resource-constrained WSN nodes. With the pre-linked mechanism, the application image size can be reduced and the underlying EJVM architecture can also be simplified. In some EJVMs [26–28], a lightweight Java OS is embedded inside. With the Java OS, the multitasking Java application can be achieved by using the Java threads. In other EJVMs [30–32], no Java OS is developed. With these JVMs, only single-task Java application can be developed.

Although a user-friendly development environment can be provided to the WSN applications by the EJVMs, the memory consumption of some EJVMs is high [27–29]. Additionally, the execution efficiency of the Java byte code is lower if compared with the machine code, thus more energy will be consumed during the run-time. Due to these reasons, it is not ideal to apply the EJVM on the high memory and energy-constrained WSN platforms.

### 4.2. Dynamic Loading Mechanism (DLM)

Dynamic loading mechanism (DLM) is another approach which can decouple the application space from the system space. With the DLM, the application can be built into a loadable module and then linked dynamically on the WSN nodes. To perform the dynamic linking, the function and variable references need to be contained in the DLM module and a dynamic linker needs to be implemented on the target device.

Currently, the DLM mechanism has been used in many WSN OSes, such as the TinyLD [13] in the TinyOS, the dynamic loader (ContikiDL) in the Contiki [33]. Moreover, the application code is built into a standard loadable ELF (Executable and Linkable Format) file in these mechanisms. The drawback of the DLM is that the application module cannot be executed directly. Instead, it needs to be resolved and linked dynamically before being executed. The advantage of the DLM is that the final executable binary is the pure machine code rather than the byte code, thus the application execution efficiency is high and less energy will be consumed.

### 4.3. New MIROS Mid-Layer Software EMIDE

In MIROS, a new mid-layer software EMIDE has been developed. EMIDE is designed with the advantages of the EJVM and DLM mechanisms, and avoidance of the drawbacks of these mechanisms. Similar to the EJVM and the DLM, EMIDE can separate the application space from the system space. However, it is designed to be both memory and energy efficient, and can thus be used even on the high memory and energy-constrained WSN nodes.

#### 4.3.1. The Design and Implementation of the EMIDE

The objectives of EMIDE determine it to be designed as follows:

*Decoupling the application space from the system space:* Like the EJVM and the DLM, EMIDE should be able to decouple the application space from the system space and function as a bridge between these two spaces.

*Machine code or interpreted code:* The byte code is used in the EJVM application while the machine code is used in the ContikiDL module. Although the byte code can be platform independent, more energy and time will be consumed during its interpretation process. Therefore, the machine code is chosen in the EMIDE. In this way, EMIDE can address the challenge of better using the tight resource-constrained WSN nodes.

*Pre-linked code or dynamic loading code:* The pre-linked application code is used in most EJVMs [26–32] while the dynamic loading code is used in most DLMs [13,33]. Compared with the dynamic loading code, the pre-linked code is smaller in size, and higher in execution efficiency. Moreover, the underlying system architectures can be simplified. Due to these reasons, the pre-linked approach is adopted in the EMIDE so that it can maintain a small memory footprint.

*Application programming language:* In most EJVMs, the language used in the application space (Java language) is different from the language used in the system space (commonly C language). As a result, two different run-time contexts should be created and this increases the complexity of the system architecture. In EMIDE, the application language is chosen to be the same as the system language. In so doing, the application code and the system code can call each other directly, thereby simplifying the system architecture.

*Support of multitasking programming in the application:* Multitasking programming is a fundamental requirement of the WSN application. In EMIDE, some mechanisms should be implemented to support the multitasking programming in the WSN application.

*Support of callback from the system space to the application space:* The callback from the system space to the application space is also needed in many cases, e.g., the low-level hardware ISR (interruption service routine) may need to be programmed in the application space. In these cases, the EMIDE should be responsible of this callback operation.

*Prevention of the faults injected from the application space to the system space:* Fault prevention is also needed in the EMIDE to prevent the faults injected from the application space to the low-level system space. The reliability of the EMIDE system can in this way be improved.

According to the design concepts above, EMIDE can be described as a mid-layer software that decouples the application code from the system code; acts as a bridge between the application space and the system space; supports multitasking programming in the application space; generates the pre-linked and machine-code application image; and is efficient in both the memory and energy cost.

**Figure 11.**
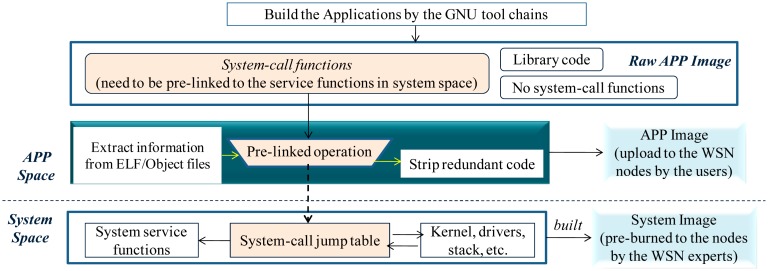
Elementary diagram of the EMIDE development process. The pre-linking is performed during the post-built process.

To implement EMIDE, several topics should be realized, including the implementation of the mechanism to pre-link the application system-call functions to the corresponding system service functions (Figure 11); the implementation of the mechanism to access the system global variables from the application program (using the “application system-call and global variable address returning” mechanism); the implementation of the mechanisms to achieve the callback operation and the multitasking programming (using the “callback registration and task registration” mechanisms); the implementation of the mechanism to prevent the application faults injected into the system space (using the “range checking and stack boundary checking” mechanisms). In the technical report [34], the implementation of the EMIDE software is presented in detail.

#### 4.3.2. Discussion

In some software like the Atmel OTAU (over-the-air-upgrade) [35], the application is not decoupled from the system, and the monolithic software image (commonly larger than 100 KB) needs to be updated for the WSN reprogramming. The reprogramming cost of this software is high and the reprogramming success probability is low. In the article [35], it is computed that in a network with 100 routers, the entire OTAU reprogramming process will take approximately 7 h to be completed for a non-secure network, 11 h to be completed for a secure network, and 14 h to be completed for a high-security network. However, if the EMIDE is applied, only the application code needs to be reprogrammed, and the reprogramming process can be completed quickly within several minutes (just hundreds of bytes need to be updated). As a result, both the reprogramming energy cost and time cost can be decreased. Another advantage of using the EMIDE is that the application code will not be built together with the system code, thus the application project becomes simple and can be reprogrammed remotely from the Internet by the webpage.

### 4.4. Performance Evaluation

In this section, the performance evaluation to the EJVMs (the full-time EJVM simpleRTJ and the tiny EJVM nanoVM), the DLM mechanism (Contiki ContikiDL and TinyOS TinyLD), the EMIDE and the Atmel OTAU is performed. All the evaluation works are done on the iLive platform (Figure 4).

#### 4.4.1. Features Comparison

The comparison of the features in the different mechanisms is shown in Table 6.

#### 4.4.2. Memory Consumption

**Figure 12.**
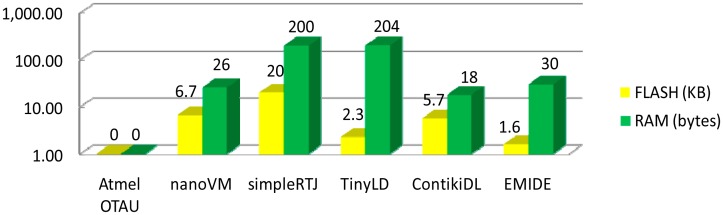
Memory cost of the EJVM, the DLM, the EMIDE and the Atmel OTAU.

The memory consumption of different mechanisms is listed in Figure 12. NanoVM has a small memory footprint, but is limited in virtual machine functionalities (Table 5). ContikiDL stores the function and variable references in the FLASH rather than in the RAM. This reduces the RAM usage, but brings down the execution efficiency. EMIDE has fewer demands on the memory resource, as it uses the pre-linked mechanism and most of the codes are implemented on the personal computer rather than WSN platforms.

#### 4.4.3. Application Code Execution Efficiency

In the ContikiDL and TinyLD, the final executed code, which is generated after the dynamic linking process, is the pure machine code. Thus, the application code execution efficiency is high. In the simpleRTJ and nanoVM, the final executed code is the Java byte code. This code needs to be interpreted, thus the execution efficiency is low. To compare the execution efficiency of the machine code and the byte code, the time between the calling of the application system-call function and the executing of the corresponded system function in the simpleRTJ and the ContikiDL is computed, and the result is shown in Table 7. From this result, it can be known that the execution efficiency of the machine code is 34.6 times higher than that of the simpleRTJ Java byte code. Therefore, less energy resource will be consumed if the machine code is used for the application image.

In EMIDE, the machine code is also used, but the access from the application system-call function to the system service function is done through an intermediate jump table, thus the application code execution efficiency is a bit lower if compared with that of ContikiDL. The execution efficiency proportion of these two mechanisms can be modeled as follows:

(3) $$P=E_{\text{EMIDE}}/E_{\text{ContikiDL}}=(10N+S)/S=1+(10N/S)$$

where *N* represents the number of the system-call functions in the application program, *10* represents the clock cycle of each entry in the jump table, *S* represents the clock cycle of the whole application code (includes the sub-functions). Provided that *S* is *1000* and *N* is *6*, then the *P* will be *1.06*.

#### 4.4.4. Application Code Size

Application code size is critical for the WSN system as it will not only determine the reprogramming success probability, but also determine the reprogramming energy and time consumption. To compare the application code size of the different mechanisms, a packet sending and acknowledgement program is used as the example, and the comparison result is shown in Table 8. In the Atmel OTAU, the application is not decoupled from the system, thus the monolithic software image needs to be reprogrammed. In the EMIDE, the reprogramming size is the minimum; because EMIDE uses the pre-linked machine code, no extra interpretation or resolving data needs to be contained in the application image. The application code size of the simpleRTJ is larger than that of the nanoVM, as simpleRTJ is full-time JVM, and a set of exception handling classes are linked inside its application image.

#### 4.4.5. Different Software Architectures to Be Built

The EJVM, the dynamic linking, and the pre-linked EMIDE can all decouple the application from the system. In this manner, a user-friendly development environment can be provided to WSN users. In Table 9, several kinds of software structures are listed. These structures have different strong points and weak points, and are appropriate to be used in different WSN contexts. The simpleRTJ can be used when the platform resources are abundant and the multitasking application programming is needed. The nanoVM can be selected when the platform resources are limited and the application task is simple. The EMIDE can be chosen when the memory and energy resources on the WSN platforms are both highly constrained.

## 5. Development of Multi-Core Hardware Platform for Energy Conservation

Energy conservation is essential for the WSN as it can prolong the nodes' lifetime and avoid the labor of bringing the nodes back for the energy recharging [2]. Currently, the sleep/wakeup mechanism used in most WSN OSes is meant to achieve energy conservation. This mechanism is effective, but not adequate for WSN proliferation.

In MIROS, the combination of both the software and the multi-core hardware technologies is used to address the challenge of energy constraint. With these technologies, a WSN node can become context aware and the energy resources can be utilized more efficiently.

### 5.1. Concepts of Using the Multi-Core Hardware Technology to Conserve Energy

Multi-core WSN node is the node on which two or more independent cores (microcontrollers) are integrated cooperatively. These cores can share the platform resources and cooperate with each other to improve the system performance. Most current WSN nodes are the single-core ones, including the Mica, the micaZ, the TelosB, the Imote, the BTnode and the EYE. However, the development of the WSN nodes trends towards the multi-core platforms. This is because some WSN challenges, such as reliability and context awareness, can be addressed better by using the multi-core platforms.

The concept of using the multi-core technology to reduce energy consumption in the MIROS derives from the experimental results that different cores are energy efficient in executing different tasks. Commonly, the cores with powerful computation ability are more energy-efficient to execute the complicated tasks while the cores with the weak computation ability are more energy-efficient to execute the lightweight tasks. In Table 10, the energy consumption of executing different tasks on the different cores is experimented. From these experimental results, it is shown that the 32-bit ARM core is more efficient in executing the complicated task, e.g., signal processing, whereas the 8-bit AVR core is more efficient at executing the simple task, e.g., sensor data collection. Due to these features, a multi-core WSN node can be developed. On this node, different types of cores can be integrated. During the run-time of a given task, the core which is the most energy efficient to execute this task can be configured to be active while the other cores can be configured to be inactive (fall asleep or be powered off). The WSN node can thus adapt flexibly to the different contexts, and the energy resource can be utilized more efficiently.

Assumed that *N* cores and *M* tasks exist on the WSN node. If these tasks are executed by the single-core nodes, the energy consumed by these nodes will be:

(4) $$E_{\text{core}-1}=\sum_{\text{i}=1}^{M}{\text{E}}_{\text{core}1-\text{task}i};\ldots \ldots {\text{E}}_{\text{core}-N}=\sum_{\text{i}=1}^{M}{\text{E}}_{\text{coreN}-\text{task}i}$$

However, if these tasks are executed by the multi-core WSN node, the energy consumed will be:

(5) $${\text{E}}_{\text{multi}-\text{core}}=\sum_{\text{i}=1}^{M}\text{min}({\text{E}}_{\text{core}1-\text{task}i},{\text{E}}_{\text{core}2-\text{task}i},\ldots {,E}_{\text{coreN}-\text{task}i})$$

Since, E_multi-core_ ≤ min(E_core-1_,E_core-2_,……, E_core-N_), the multi-core node is more energy-efficient.

### 5.2. Implementation of the Multi-Core WSN Nodes

The cores on the multi-core node can be classified into two kinds: the working cores and the management core. The working cores are commonly very different from each other so that the multi-core node can adapt to the diverse contexts better. The management core is ideally the one which is low in the energy cost and high in the reliability; its responsibility is to configure the working cores to run in different statuses (active or inactive). With this configuration, the multi-core node can work in different modes and become context aware to the varied application contexts.

**Figure 13.**
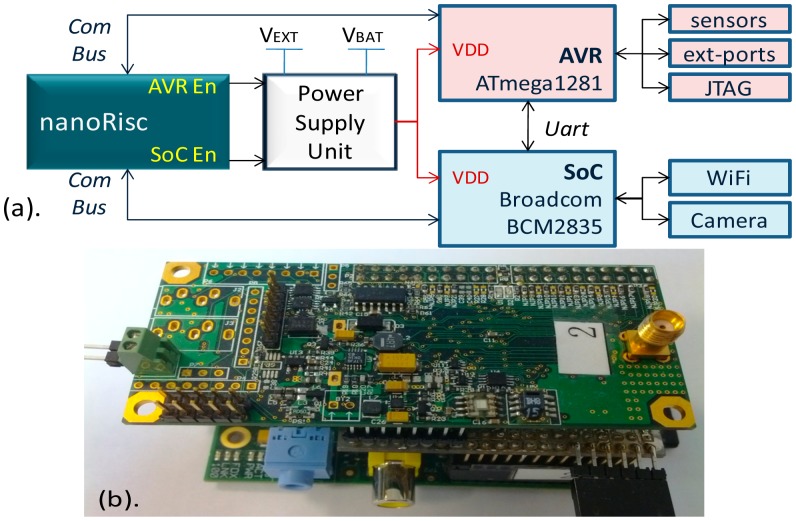
(**a**) Block diagram of the MiLive. (**b**) Prototype board of the MiLive.

In Figure 13, the block diagram and the circuit board of the multi-core node MiLive are shown. MiLive consists of three cores: the 4-bit ultra-low power NanoRisc core, the 8-bit low power AVR ATmega1281 core, and the 32-bit powerful SoC BroadcomBCM2835 core. The NanoRisc acts as the management core. It can control the Power Supply Unit (PSU) to configure the working cores to run in a different status. The status of a working core can be active or be inactive. The core in the inactive status can either be powered off or fall asleep. The power off operation is performed when the inactive period is short while the sleeping operation is performed when the inactive period is long. By means of this multi-core platform and the configuration mechanism, the MiLive node can self-adapt to the different application contexts and be highly energy efficient, as seen in Table 11.

### 5.3. Performance Evaluation

**Figure 14.**
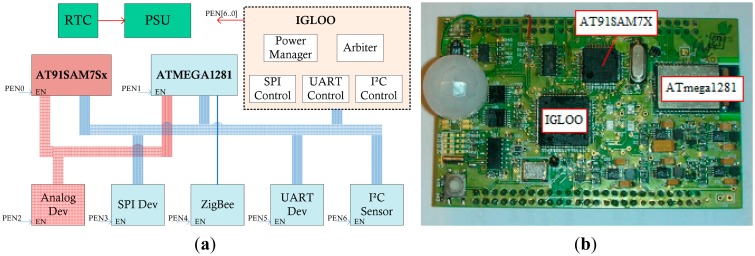
(**a**) Block diagram of the multi-core node EMWSN. (**b**) Prototype board of the multi-core node EMWSN.

To evaluate the energy conservation performance, the multi-core node EMWSN is used (Figure 14). EMWSN consists of three cores: the 8-bit low power AVR core (ATmega1281), the 32-bit low power ARM core (AT91SAM7Sx) and the good leading-time ultra-low power iGLOO FPGA [36]. The iGLOO FPGA acts as the management core. It can configure the EMWSN to work in different modes without the change of the wired connection.

According to the experimental results in the previous Table 10, the lifetime of different WSN nodes can be calculated. If the node is powered by a pair of AA batteries and the temperature and light sensors sample every 3 min, the theoretical lifetimes of the multi-core EMWSN node, the single-core AVR node, the single-core ARM node and the single-core TelosB node will respectively be 1270 days, 825 days, 382 days and 945 days. From this result, it can be concluded that the multi-core platform is more efficient in the energy consumption.

## 6. Multi-Core Hardware Platform for the Reliability Improvement

In past research experience, it was proved that 30% to 50% Live nodes [37] failed after being deployed for two months, although these nodes worked properly for long periods when tested in the lab desktop. Since most WSN nodes are deployed in hostile environments where they are difficult to be brought back for repairing, the improvement of node reliability becomes a key research challenge. Currently in the MIROS, this multi-core hardware technology is also used to improve the node reliability.

One way of using the multi-core hardware technology to improve the node reliability is the usage of the redundancy approach. For example, on the EMWSN node (Figure 14) the sensor peripherals can be managed by both the AVR core and the ARM core. Commonly, the AVR core is in charge of the sensor data collection work. In the case that the AVR core fails, the ARM core can substitute it to continue this task, thereby improving node reliability.

In addition to the redundancy method, another way to improve the node reliability is to use a more reliable and ultra-low power core (management core) to manage a more powerful but less reliable and high power core (working core). Currently, this approach has been used on the multi-core node iliveT. On the iliveT, two cores are equipped: the 8-bit working core AVR ATmega1281 and the 4-bit management core nanoRisc. The AVR core is more powerful and can be used to perform WSN tasks, and MIROS runs on this core. The nanoRisc is low-end and cannot be competent for most WSN tasks. However, it is ultra-low power and highly reliable [38], and is thus ideal to be used as the management core to configure the work modes of the working core.

The following is an example scenario: the working core AVR wakes up every six hours to collect the environmental data, and then transmit the data packet to the local server. To achieve this task, a periodical six-hour timer is set on the management core nanoRisc. Every time the periodical timer is fired, the power will be supplied to the AVR core. Once powered, the AVR core starts collecting the environmental data, and then sends a handshaking signal from the GPIO ports to the nanoRisc. If the handshaking signal is received by the nanoRisc within a given time, the AVR core can be powered off or fall asleep. If not, the exception may occur on the AVR core. In this case, the nanoRisc will restart the AVR core by the power control (power off first, and then power on again). In this manner, the reliability of the data collection task can be improved.

The advantage of using this multi-core technology on the iliveT is that the reliability of the node will no longer be determined by the less reliable working core, but rather be determined by the highly reliable management core. In past experiments from two years ago, three single-core iLive nodes were deployed in the garden of ISIMA, and one of them failed only after working for two weeks. However, no nodes have failed thus far after the multi-core iliveT nodes were applied (and have been working properly for more than two years; the environmental temperature ranges from −15 degree to 35 degree). This result proves that the multi-core technology used on the iliveT is effective for reliability improvement.

Besides the improvement to the node reliability, the multi-core technology can also decrease the energy consumption of the iliveT nodes. If the single-core iLive node (only the AVR core) is used, the AVR core needs to fall asleep during the idle period. However, if the multi-core iliveT is applied, the AVR core can be powered off when it is idle, and be woken up later by the nanoRisc. Since the sleeping current of the nanoRisc core is much smaller than that of the AVR core (nanoRisc: 3.3 μA. AVR core: 130 μA [39]), many energy resources can be conserved if the idle period is long. Although two cores are equipped on the iliveT, the manufacture cost of the iliveT does not increase significantly. This is because nanoRisc only needs to manage the working core, and can thus be chosen to be a low-end and inexpensive microcontroller.

## 7. Miscellaneous

### 7.1. Inter-Process Communication

Inter-process communication (IPC) is needed for the data exchange among the multiple tasks. In MIROS, the message queue is used for the IPC implementation, and the MIROS IPC is used not only for the communication among the tasks inside one core, but also for the communication among the tasks on different working cores, e.g., the communication between the AVR core and the ARM core in Figure 14. All the IPC operations in the MIROS are realized through the universal interfaces “*send*” and “*recv*”. In this manner, not only the programming complexity on the WSN nodes can be simplified, but also the application porting among the different cores can be eased. Note that MIROS commonly runs on the working cores other than the management core. Thus, the MIROS IPC is only used for the communication among the working cores. As for the communication between the management core and the working core, it is achieved through the signal change on the GPIO ports.

### 7.2. Development of a New Debugging Approach by Using the Multi-Core Platform

At present, the serial port approach is commonly used for the debugging in WSN. The values of the inspected variables can be sent to the personal computer through serial communication. The drawback of this approach is that the overhead is high, thus the execution of the regular code will be influenced by the debugging process. Moreover, the serial transmission speed is limited, and this may cause the transmission of the debugging data to overflow.

In addition to the serial port debugging, the breakpoint setting is another method. This method is useful when the execution process is logically sequential, but it is not effective when the execution process is concurrent. In addition, the system run-time will stop once a breakpoint is met, and this can cause some underlying problems, e.g., if the ZigBee [40] protocol is used in the WSN, the nodes which stop execution during the debugging process will be regarded as being lost from the network. In this case, the network coordinator will delete this node, and then the real-world debugging on this node can no longer be performed.

**Figure 15.**
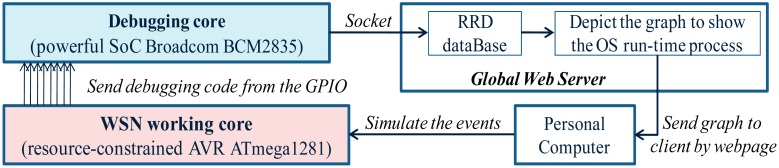
New debugging approach by using the multi-core technology.

In the MIROS, a new debugging method is developed by using the multi-core hardware technology. In Figure 15, the diagram of the multi-core debugging system is depicted. A less powerful resource-constrained working core AVR ATmega1281 is connected to a more powerful resource-abundant debugging core ARM BCM2835. The working core is in charge of executing the WSN tasks while the debugging core is in charge of executing most of the debugging tasks. The debugging burden on the working core can therefore be eased significantly. As a result, the execution of the regular code on the working core will be less influenced by the debugging process.

Two cores communicate with each other through the GPIO (global input and output) interfaces. The GPIO port is chosen not only because the data transmission speed from this port is high, but also because the data transmission overhead from this port is low. With this communication mechanism, the data transmission overflow problem can be avoided. In addition, the system run-time on the working core will not halt during the debugging process. Consequently, the real-world debugging can be better achieved. This multi-core debugging method is presently used for monitoring the run-time status of the WSN nodes remotely from the Internet. As illustrated in Figure 15, every time the working core makes an action, it will send the debugging data corresponding to these actions to the debugging core. The debugging core buffers, parses and forwards these debugging data to the web server. Later, this data information will be converted into the graph and sent to the users through the webpage.

### 7.3. Comparison of Different Embedded OSes

In Table 12, a comparison among the different embedded OSes is shown. In the TinyOS, Contiki, SOS and openWSN, the preemption is not enabled and the real-time performance is poor. In the mantisOS and simpleRTJ, the preemption is supported, but no real-time scheduling algorithm is implemented. As a result, the real-time deadlines can still not be guaranteed. In the FreeRTOS and the uCOS, the fixed-priority preemptive scheduling and the RMS scheduling are achieved respectively; these two OSes are the popular RTOSes.

In the FreeRTOS, four kinds of memory allocators are realized, and an appropriate one can be selected depending on the application contexts. However, the memory fragments cannot be assembled in these allocators. OpenWSN implements an open-source fully standards-based protocol stack which includes the RPL, the 6LowPAN and the IEEE802.15.4e [41]. With the IEEE802.15.4e, an ultra-low power and highly reliable network can be established. FreeRTOS and the uCOS are the general-purpose OSes while the others are the WSN-dedicated OSes. Compared with the general-purpose OSes, the WSN OSes are typical in that they target at keeping small memory footprint, low execution overhead, low energy consumption as well as high reprogramming performance.

### 7.4. Code Size of Different Components in MIROS

**Figure 16.**
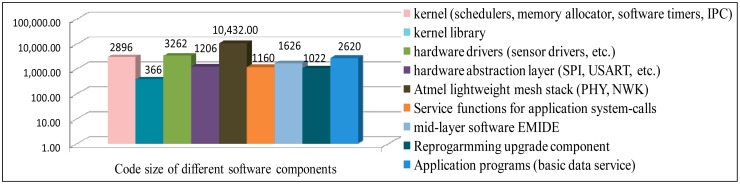
Code size of the different MIROS components on the iLive platform.

Currently, two kinds of network stacks, the Atmel IEEE802.15.4 [42] and the Atmel lightweight mesh stack [43], have been ported to the MIROS. In Figure 16, the code size of different components in the MIROS is shown. If the SFL allocator is applied, the size of the MIROS kernel is 2896 bytes. If the Atmel mesh stack is used, the size of the software firmware is 24,590 bytes. If the Atmel IEEE802.15.4 stack is used, the size of the software firmware is 53,234 bytes. These results prove that the MIROS system is suitable to be used on many popular WSN platforms (BTnode, IMote, SenseNode, TelosB and T-Mote Sky, *etc.*) to provide the basic sensing data services.

## 8. Conclusions and Ongoing Work

In this article, a real-time, memory-efficient, energy-efficient, user-friendly and fault-tolerant WSN OS MIROS is designed and implemented. MIROS achieves the real-time scheduling with less RAM cost. With this feature, the real-time WSN applications are feasible to be executed on the low-end high memory-constraint WSN nodes, e.g., after the MIROS is applied, the memory-constrained iLive nodes can be used to run the time-critical industrial engine control tasks. Besides this feature, MIROS also shows the advantages in having a long lifetime, being fault-tolerant and supporting efficient remote reprogramming. Consequently, the WSN nodes become practicable to be deployed in the harsh environments where the human-labor maintenances (e.g., the effort to bring the nodes back after the deployment for the power recharging, the application reprogramming and the fault reparation) are difficult. Furthermore, MIROS provides a user-friendly application development environment. In this manner, the WSN proliferation can be promoted to more application domains. Typically, MIROS can be applied in the contexts with the following rigorous conditions: (1) nodes are equipped with the low-end microcontrollers, and are constrained in the memory and energy resources; (2) a real-time guarantee is required by some tasks; (3) the deployment environment is harsh so that it is difficult to re-collect the nodes after they are deployed. As for the ongoing work, MIROS will focus on the following topics:

*Finite-state-machine MIROS:* To improve the software reliability further, all the system services in MIROS will be developed by using the finite-state-machine programming style. Every time the code related to a state is executed, the execution result will be checked. If the result is incorrect, the roll-back recovery will be performed. Only when the result is validated, can the execution transit into the next state. An execution error can thus be limited to spread widely, and the run-time failure probability can be decreased.

*Establishment of the intra-communication network among the multiple cores:* To simplify the management of the multi-core WSN platform, the communication among the multiple cores on the multi-core platform will be operated as the communication among the nodes in a network. For example, the communication among the cores on the multi-core node can be achieved in the networking modes: P2P (point to point), P2M (point to multi-point) and M2P (multi-point to point). Moreover, the “channel/pipes” concept (similar to the “socket/ports” concept in the TCP/IP protocol) will be applied in the MIROS IPC to abstract the low-level hardware communication details.

*System on Chip (SoC) technology:* Currently, different multi-core WSN nodes (EMWSN, MiLive, iliveT, *etc.*) have been developed to meet the different application contexts. This development method is complicated in the manufacture process and high in the maintenance cost. In the next generation of the multi-core hardware development, the SoC technology will be used. With the SoC, the working modes of the multi-core nodes can be reconfigured by uploading different firmware. Once reconfigured, a node can change the modes and adapt to the new application context. In so doing, one integrated multi-core node can be used to support different kinds of WSN applications. As a result, the manufacture cost can be reduced and the software development cycle can be minimized.

*Parallel computing to improve the real-time performance:* The multi-core technology is presently used to reduce energy consumption, to improve system reliability and to realize the new debugging approach. In future work, this technology will also be used to improve the system real-time performance. In the case that two or more hard real-time tasks are triggered simultaneously, these tasks will be distributed onto different cores and be processed concurrently. The deadlines of the real-time tasks can therefore be better guaranteed.

*AADL for the OS simulation and verification:* The AADL (Architecture Analysis and Design Language) is a model-based engineering language used to analyze the reliability, availability, timing, responsiveness, throughput, safety and security of the software and hardware architectures [44]. In the ongoing work, the AADL will be used in MIROS to achieve the objective of developing a formal verifiable, highly reliable, fail-detectable and self-recoverable system.

## Figures and Tables

**Table 1.** Date memory cost of the event-driven and multithreaded schedulers in different OSes.

**WSN OSes**	**Scheduling Mechanism**	**Data Memory Consumption**		**Scenario**	**Data Size (bytes)**	
		**Event-Driven**	**Multithreading**		**Event-Driven**	**Multi-Threading**
TinyOS	Event-driven	8 + 1*L_SQ_	N/A	L_SQ_ = 15, N_PCB_ = 12 N_TCB_ = 8, N_THRD_ = 5 N_TCB2_ = 15, N_THRD2_ = 12, S_STK_ = 120	23	/
	TOSThread	N/A	22 + 16*N_TCB_ + Size(STK)		/	750
Contiki	Event-driven	10 + 6*L_SQ_ + 8*N_PCB_	N/A		196	/
	Multithreading	N/A	8 + 8*N_TCB_ + Size(STK)		/	672
SOS	Event-driven	62 + 18*L_SQ_ + 24*N_PCB_	N/A		620	/
mantisOS	Multithreading	N/A	40 + 22*N_TCB2_ + Size(STK2)		/	1,810
MIROS	Event-driven	4 + 4*N_PCB_	N/A		52	/
	Multithreading	N/A	4 + 8*N_TCB_ + Size(STK)		/	668

**Table 2.** Clock cycles of the event posting and task dispatching primitives in different schedulers.

**Event-Driven Scheduling Primitives**	**Clock Cycles (AVR ATmega1281)**					**Clock Cycles of One-Byte Memory Copy**
	**TinyOS**	**Contiki**	**SOS**	**MIROS**	**mantisOS**	
Event post	10	28	39	17	N/A	8
Task dispatching	46	36	53	26		

**Table 3.** Clock cycles of the thread switch and thread selection primitives in different schedulers.

**Multithreaded Scheduling Primitives**	**Clock Cycles (AVR ATmega1281)**				
	**TinyOS TOSThread**	**Contiki Multithreading**	**SOS**	**MIROS**	**mantisOS**
Thread switch	77	93	N/A	102	99
Selection of next thread	26 (RR scheduling)	22 (RR scheduling)		5 + 9n (RMS scheduling)	72 (RR scheduling in multi-level queues)

**Table 4.** Feature comparison of the different allocators.

**Allocation Mechanism**	**Allocation Status**	**Execution Efficiency (Evaluated by the Cost of Clock Cycles)**				**Fragments (Internal or External)**	**Memory Utilization Efficiency**
		**Allocation**		**De-Allocation**			
		**Clock Cycles**	**Cost**	**Clock Cycles**	**Cost**		
Contiki *SFL*	N/A	23 + 12*F	Low	26 + 10*F	Low	External	Low
SOS *SFL*		34	Low	38	Low	Both exist	Low
MantisOS *SF*		52 + 15*L	Medium	37 + 16*L	Medium	External	Medium
MIROS *SFL* (heap-extendable)	Regular	36	Low	32	Low	External	Medium
	In extended heap	45 + 16*L	Medium	43 + 18*L	Medium		
MIROS *SF* (proactive defragmentation)	N/A	36	Low	32 + 20*S	High	External, but can be assembled	High
MIROS *SF* (reactive defragmentation)	Regular	56 + 12*L	Medium	45 + 15*L	Medium	External, but can be assembled	High
	Fragments need to be assembled	92 + 12*L + 15*S	High	45 + 15*L	Medium		

**Table 5.** Feature comparison of the current popular EJVMs.

**EJVMs**	**Java APP Linking Model**	**Exception Handling**	**Garbage Collection**	**With Stand-Alone JavaOS**	**Multitasking Java Application Support**	**Code Size**
TinyVM	Pre-linked	Support	No	Yes, multithreaded Java OS	Yes, by Java threads	about 10 KB on the RCX
DarjeelingVM	Pre-linked	Support	Support	Yes, multithreaded Java OS	Yes, by Java threads	about 15 KB on the AVR
SimpleRTJ	Pre-linked	Support	Support	Yes, multithreaded Java OS	Yes, by Java threads	18–23 KB
Squawk VM	Pre-linked	Support	Support	No, can run without a Java OS	Yes, by application isolation mechanism	about 80 KB on the SunSPOT
nanoVM	Pre-linked	No	Support	No	No, can run only single-task Java APP	<10 KB for AVR ATmega8
Jwik	Pre-linked	No	No	No	No	<10 KB
Java Card VM	Pre-linked	Support	No	No	No	<15 KB

**Table 6.** Feature comparison of the EJVM, the DLM, the EMIDE and the Atmel OTAU.

**Features**	**EJVM (simpleRTJ, nanoVM)**	**DLM (TinyLD, ContikiDL)**	**EMIDE**	**Atmel OTAU**
APP decoupled from system	Yes	Yes	Yes	No
APP programming language	Java	C	C	N/A
APP image format	Pre-linked byte code	CELF and microExe	Pre-linked machine code	N/A
Flexibility of APP image	Medium	Well	Medium	N/A
APP multitasking programming	Supported, by Java threads	N/A	Supported, by the registration mechanism	N/A
Mechanism to call from the APP to the system	By Java native mechanism	By dynamic linking	By pre-linking	N/A
Mechanism to callback from the system to the APP	By the VM byte code interpreter	N/A	By the callback registration mechanism	N/A
Exception handling support	Supported in simpleRTJ	N/A	Fault prevention mechanism is implemented	N/A
Garbage collection	Yes	N/A	N/A	N/A

**Table 7.** Comparison of the application code execution efficiency.

**APP Code Type**	**Mechanism**	**Execution Status (Execution in Loop for 2,000,000 Times)**				
		**Voltage (V)**	**Current (mA)**	**Time (S)**	**Energy Cost (mJ)**	**Proportion**
Machine code	ContikiDL	3	10.5	14	441	1
Java byte code	simpleRTJ	3	10.5	485	15,277.5	34.6

**Table 8.** Comparison of the reprogramming code sizes in the different mechanisms.

**Application Features**	**TinyLD**	**ContikiDL**	**simpleRTJ**	**nanoVM**	**EMIDE**	**Atmel OTAU**
Code format	microExe (compacted ELF)	Compacted ELF	Pre-linked Java bytecode	Pre-linked Java bytecode	Pre-linked machine code	Monolithic software
Code size (bytes)	612	768	2,472	876	182	114,786

**Table 9.** Features of the different software structures.

**Software Architectures**	**Multitasking APP Programming**	**Real-Time Scheduling**	**Garbage Collection**	**Exception Handling**	**Code Size (KB)**
nanoVM (Feature-limited)	No	No	Yes	No	7.8
simpleRTJ	Yes, by Java threads	Soft RT	Yes	Yes	22.3
nanoVM + MIROS	Yes, by Java threads	Yes	Yes	No	11.2
simpleRTJ + MIROS	Yes, by Java threads	Yes	Yes	Yes	23.8
EMIDE + MIROS	Yes, by registration mechanism	Yes	No	No	4.3

**Table 10.** Energy consumed by executing different tasks on different cores.

**Tasks for the Measurements (Voltage: 3 V)**	**32-bit ARM AT91SAM7Sx**			**8-bit AVR ATmega1281**		
	Current (mA)	Time (ms)	Energy (mJ)	Current (mA)	Time (ms)	Energy (mJ)
Temperature and light sensor sampling task	22.1	896	59.4	15.9	900	42.9
Signal processing task (pure instruction execution)	19.7	5.0	0.296	9.9	268	7.96
Flash programming (10-byte)	20.9	6.0	0.376	16.3	10.1	0.49
Sleep	0.2	N/A	N/A	40 μA	N/A	N/A

**Table 11.** Different working modes of MiLive.

**Working Modes**	**Different Core Status**			**Strong Points of This Mode**	**Run-Time Contexts to Be Applied**	**Equipment That Is Available in This Mode**
	**NanoRisc**	**AVR**	**SoC**			
AVR-core	Active	Active	Inactive	More energy-efficient in transmitting small-sized, low-rate messages and in processing the simple tasks	When executing the scalar WSN tasks	Sensors, IEEE 802.15.4 wireless medium, *etc.*
SoC-core	Active	Inactive	Active	More energy efficient in transmitting large-sized, high-rate messages and in processing complicated tasks.	When executing the WSN multimedia tasks	IEEE 802.11 WiFi, camera, *etc.*
Hybrid	Active	Active	Active	Can self-adapt to different contexts. Highly context aware	Mix of the above two	Both of above
Sleep	Active	Inactive	Inactive	Ultra low energy consumption	When node are idle, to conserve the energy	N/A

**Table 12.** Feature comparison of the different embedded OSes.

**WSN OSes**	**Titles**					
	**Scheduling Model**	**Real-Time Supported**	**Memory Allocation**	**Decoupling of APP from System**	**Network Protocols**	**Energy Conservation**
TinyOS	Event-driven	No	Static	No	TYMO, 6LoWPAN, Active message, TDMA, IEEE 802.15.4, *etc.*	Sleep/Wakeup
TinyOS with TOSThread	Hybrid	Not well, event-driven scheduling in native layer		Yes, by dynamic linking		
Contiki	Event-driven	No	Dynamic SFL	Yes, by dynamic linking	uIP, Rime, ContikiRPL, 6LowPAN, *etc.*	Sleep/Wakeup, controlled from the App program
Contiki with multithreading	Hybrid	Not well, event-driven scheduling in native layer				
SOS	Event-driven	No	Dynamic SFL	Yes, by position independent code	Message, AODV, ICMP, *etc.*	Sleep/Wakeup
MantisOS	Multi-threaded	Not well, the RR scheduling is used	Dynamic SF	No	Comm	Sleep/Wakeup
openWSN	Event-driven	No	Static	No	CoAP, HTTP, UDP, TCP, RPL, 6LoWPAN, IEEE 802.15.4e	Tight time synchronization by IEEE 802.15.4e
simpleRTJ	Multi-threaded	Not well, the RR scheduling is used	Static	Yes, by virtual machine	N/A	Sleep/Wakeup
uCOS	Multi-threaded	Yes, preemptive real-time RMS scheduling	Dynamic SFL	N/A	μC/TCP-IP	Sleep/Wakeup
FreeRTOS	Multi-threaded	Yes, supports both preemptive and cooperative scheduling	Four approaches	N/A	Thread-aware UDP	tickless mode for low power applications
MIROS	Hybrid	Yes, preemptive real-time RMS scheduling	Improved SFL and SF	Yes, by pre-linking EMIDE	Atmel IEEE 802.15.4, Atmel lightweight mesh stack	Sleep/Wakeup and Multi-core context aware mechanism
